# Logged in While Locked Down: Exploring the Influence of Digital Technologies in the Time of Corona

**DOI:** 10.1177/1077800420960176

**Published:** 2021-09

**Authors:** Kathrine Liedtke Thorndahl, Lasse Nørgaard Frandsen

**Affiliations:** 1 Aalborg University, Denmark

**Keywords:** corona, autoethnography, digital technologies, collage

## Abstract

Based on a collaborative endeavor, we present an autoethnographic textual collage of three fictionalized narrative accounts produced as a result of an amalgam of personal experiences encountered during the corona pandemic. The narrative accounts serve as illustrating examples of and critical reflections on how the pandemic and the increased use of digital technologies during the pandemic have affected the experience of reality and (trans)formed thinking and social relations alike. The crisis invites us to reflect on how the current confinement saturated with the ubiquitous presence and influence of digital technologies can be used as an occasion for large-scale reflections.

The date is March 17, 2020. It’s a Tuesday. The corona pandemic is wreaking havoc across all continents except in Antarctica. Penguins apparently are immune to its deadly effects. Europe has taken a hard blow, especially Italy, where more than 2,900 people have died between now and February 23 when they started counting. Many countries, including Austria, Spain, and France, have declared a national state of emergency and have forbidden people to leave their homes.

“We are at war, certainly a health war,” the French president Emmanuel Macron stated in a televised speech and added in martial language:

We are not fighting against an army or against another nation. But the enemy is there, invisible, elusive, advancing. And this requires our general mobilization.

After delivering his address, in which Macron announced a 30-day nationwide lockdown while explaining in unambiguous terms the measures put in place to try to contain or, at the very least, alleviate the spread of the virus, the TV station played La Marseillaise, the national anthem, as if to finally drive home the message and to underscore the severity of the situation by instilling a kind of solemn sobriety in its viewers. With its forceful lyrics and vivid use of military references, La Marseillaise mirrors the words of the president and seems to fit the occasion perfectly.

## Logged in While Locked Down

As a consequence of the lockdowns put in place by governments in numerous countries around the world to prevent the spread of coronavirus, many of the activities that many of us used to take for granted, like traveling and going out and hugging each other, suddenly became impossible. Therefore, a great many of us have had to adapt to new and changing conditions and to adjust the way we engage with the world in accordance with the new demands of a new reality. Thus, in addition to fighting the virus itself, we now also face the additional challenge of having to make sense of the world from a new perspective and by alternative means. To a great extent, this new perspective and the kind of sensemaking that results depend on the use of various digital technologies in the form of social media such as Facebook, Instagram, and Twitter as well as various digital online communication platforms such as Skype, Teams, and Zoom. Importantly, when these digital technologies are not merely seen as neutral transmitters of information but rather as performative infrastructures (cf. Gillespie, 2010), they become *mediating* agents in their own right that not only facilitate processes of sensemaking but also shape the performance of social acts (van Dijck, 2013).

To try to cast light on the implications of seeing digital technologies as mediating agents, we present three autoethnographic accounts (Ellis, 2009; Ellis & Bochner, 2000) to explore how digital technologies are employed and how they function in particular ways to influence and shape how we make sense of the “Self, the Other, and the World” (Markham & Harris, 2020) during the corona pandemic, as part of the collaborative project this special issue addresses. To achieve this aim, we invite readers to join us on an experimental journey through the uncharted territory of daily life by describing and reflecting on three narrative accounts that serve as examples intended to illustrate that and how life in the time of corona is heavily influenced by the endemic presence and use of digital technologies.

## Puzzles, Premises, and Procedures

If reality itself, as well as the research meant to describe and explain it, used to feel like a puzzle, albeit a puzzle with a few missing pieces, because of the corona pandemic, it now feels as if this puzzle has suddenly been shoved off the table. To make matters worse, in passing through the liminal space between the solid surface of the table and the ground beneath it, the individual pieces of the puzzle seem to have somehow transformed into something that can no longer be recognized as pieces of a puzzle at all—like a ball, a lemon, and a stapler in addition to a hotchpotch of numerous other and utterly incompatible things. No longer are we able to construct nor even imagine how this strange and motley collection may be combined and made to resemble a puzzle nor in the least the idyllic scenery that the pieces of the puzzle used to be able to convey if assembled correctly. It follows that the puzzle metaphor no longer seems to suffice to adequately convey anything meaningful about the nature of reality nor about the function of research nor about the latter’s ability to accurately describe the former or not. Therefore, instead of adhering to the realist and objectivist understanding conveyed by the puzzle metaphor that reproduces representationalism and maintains that meaning-making happens through the logic of either/or due to the particular structure of language, we need an alternative, and so we exchange the puzzle metaphor for one that seems better suited for our purposes: the metaphor of *collage*.

In the context of qualitative inquiry especially the kind informed by poststructuralist notions, collage is used to refer to a particular representational form in which multiple different types of content and materials are combined to make meaning in a way that relies on juxtaposition and difference rather than coherence and sameness (Butler-Kisber, 2008). As such, the collage metaphor invokes images of working with gaps and overlaps, holes and silences, absence, and excess while insisting on the idea of relationality and forms of inquiry that are mindful of the multivocal and nonlinear representational potential of research (Kangas et al., 2018). Thus, the representational form of collage is closely related to the methodological approach of bricolage (Denzin & Lincoln, 2000; Kincheloe, 2001; Markham, 2005) as both of them are “fluid, eclectic, and creative” (Rogers, 2012, p. 5) and result in a “critical, multi-perspectival, multi-theoretical and multi-methodological approach to inquiry” (Rogers, 2012, p. 1). It follows that the texts that result from using a bricolage approach or from presenting one’s findings in the form of a collage are not merely neutral and innocent means of communication, far from it. As with other approaches to inquiry, they create the worlds we study (Denzin, 2016; Law, 2004).

Contrary to a puzzle, a collage is an assemblage of different components that are glued together to form a whole, albeit not one that portrays a complete and fully coherent image. The lack of such an image, however, does not mean that the collage metaphor is devoid of explanatory power. Because thinking with the collage metaphor encourages a problematization of the assumptions of linearity (Butler-Kisber, 2008) and representationalist ideals embedded in realist ontologies and objectivist epistemologies, it is well-positioned to leave an impression and to affect the reader and the world in profound ways. Indeed, because of the inherent ambiguity that remains present in collage, this representational form “provides a way of expressing the said and the unsaid, and allows for multiple avenues of interpretation and greater accessibility” (Butler-Kisber, 2008, p. 268). In a similar vein, Markham (2005) contends that qualitative methods of inquiry and representational forms, including but not limited to collage, fragmented narrative, pastiche, and layered accounts, also hold the potential to function “politically to encourage multiple perspectives” (p. 814).

The collage we present in this paper is a layered account (e.g., Rambo Ronai, 1995) consisting of three fictionalized and severely condensed autoethnographic narrative accounts in addition to several snippets of interpretive text. The three fictionalized accounts are inspired by the complex multitude of personal and shared experiences that we encountered during the first months of the corona pandemic.

More specifically, we use the three narrative accounts to describe and reflect on the intellectual and emotional possibilities and limitations of the new (virtual) reality created by the corona pandemic. Paraphrasing Bernard Stiegler (2020), the narrative accounts are meant to provide an invitation for reflection on how the current confinement saturated with the ubiquitous presence and influence of digital technologies can be used as an occasion for a large-scale reflection not only on our relation to these technologies but also on the possibility and the need to effect positive changes that may lead to a more socially just and ecologically sustainable future.

Thus, while the primary focus of our paper is on exploring the way life in the time of corona is influenced by the use of digital technologies, we also want to signal in the direction of larger social issues, global as well as local. Doing so situates our paper in conversation with several other contributions in this special issue among them Torres (2020), Irwin (2020), and Zheng (2020), all of whom grapple with similar concerns about how it may be possible to promote an affirmative agenda emphasizing kindness, social justice, and ecological sustainability in the face of hostility, despair, and disconnect. To that end, we try to illustrate how the use of digital technologies has affected the experience of reality, that is, the world, and (trans)formed thinking as well as social relations, that is the Self and the Other, in the time of corona by cultivating the kind of autoethnographic writing that may be characterized as “an ‘autoethnography to come’ that is endlessly expansive, inventive, and creative” (Gannon, 2018, p. 21).

By employing this form of writing, we hope that our paper will be able to fulfill an additional purpose: To be a Munchian scream to the world that will make us pause, think, and feel; to make us start, in other words, to pay attention to the constant destruction caused to a great extent by the way we choose to live.

## We Are Not I

Even though there are two of us, and even though the three narrative accounts we present were brought about as a result of a highly cooperative effort of mutual engagement in which we used collaborative writing (Gale & Wyatt, 2010) and writing as a method of inquiry (Richardson & St Pierre, 2018) to tease out evocative descriptions (Tyler, 1986) of mundane experiences, we deliberately use the singular first-person form throughout the narratives.

As we deliberately refrain from identifying the narrating “I” in terms of gender, age, and other identity markers, we end up with a universal, first-person narrator, purposely invented and described as a generic composite character that could be anyone. In fact, you can call them “Mary Beton, Mary Seton, Mary Carmichael or by any name you please—it is not a matter of any importance” (Woolf, 1929/1977, pp. 8). What is of some importance, however, is the fact that just as Virginia Woolf and her narrator are separate entities, we are not the narrator in the narrative accounts we present. While we might struggle with the same issues as the narrator, none of us can be said to be or identify directly with the narrator, just as the experiences described by the narrator cannot be said to mirror ours as these descriptions are also composite structures inspired by multiple different events.

In keeping with this particular understanding of the role and status of the narrator and the narrative accounts, we do not provide authoritative interpretations of what each of the narratives means. And so while we cannot deny the fact that we most certainly try to make the reader see things from our perspective, we also heed Denzin’s (1997) call for authors to deliberately relinquish their interpretive authority and to simultaneously leave behind the idea that it is possible to produce objective descriptions of an external and independently existing reality.

## W(h)ining and Dining

Bling. I get a message with a link on Facebook. Open link. A video. Play. Watch. Watch again. I cannot make out what it is. I move closer to the screen. Squint. Still unable to make sense of the moving images, I reply to the message with a question: “What is this?” It takes me yet another try and an explanation before I understand what it is I am looking at. Incubation time. An update from downtown Kampala in Uganda. In the video, a massive crowd of people is jam-packed around a truck. Three men standing on the truck bed are throwing white bags of something randomly into the crowd. The people scream and shout, pushing and struggling to get their hands on the white bags. No social distancing there. It looks chaotic, as if the situation is out of control.

Once I get the picture, I am immediately infected. No need for a test. I know it. I feel the virus spreading through me until that video seems to be all I can think of. I respond in affect. I want to create a chain of infection. Copy. Paste. Share Now (Public). I honestly expect it to go viral in a matter of minutes when I share it on my Facebook page. Let everybody in my network get infected, I think to myself. To my surprise, however, just seven of my 486 friends react to the video posted under the caption, “Puts things in perspective.” Why don’t they care? I ask myself in despair. At that point, it occurs to me that it is not so much the situation in and by itself that really bothers me. It is not the video of the situation either, nor is it the first or second sharing of the video of the situation that gets to me. Instead, it is the lack of response I got when sharing the sharing of the video of the situation that strikes me as absurd.

Later that same day, the symptoms seem to grow stronger. While still in shock, I call up a friend of mine. I feel frustrated and powerless. Every cell in my body aches with these feelings as if they were cuts and bruises inflicted on my limbs. I need to share. The video. My feelings. We connect to the same line. Online. There she is occupying 90% of the screen. I’m in the corner, facing myself. But if my doppelgänger is there, that means my friend’s doppelgänger must be here right next to me?! I turn to check, but I don’t see her. All through our conversation, I notice how I am extremely preoccupied with the moving image of myself, and of how my eyes keep wandering to the lower right corner of the screen to meet her eyes that are my eyes. Am I in love with my own image? Like Narcissus? I wonder. Hardly. But I cannot deny that it draws me in, my own image, that is.

After exchanging a few brief niceties, I tell my friend how I received the video via another new friend of mine. He got it from his girlfriend, who lives in Kampala. I feel my temperature rise as I tell her the story. When I finally stop to breathe, I scan her face for signs of resonance. But I find no such signs, and so while *I* am trying with all my might to come to terms with the images of that crowd, my friend seems unaffected. Indeed, instead of empathizing with my feelings of hopelessness and frustration about the present state of the world, it is as if she is actually *enjoying* the situation, as if she is finding *joy* in the small details of home-bound life, as she sits there in front of her screen, sipping white wine after a day of watching Netflix. She looks at me with eyes smiling and replies to my story with a question:

Do you have a glass of wine? That’s my medication these days.

I stare right back at her.

“I mean, I know what you mean, but here we just try to make the best of it. It’s actually kind of cozy,” she adds.

At that moment, I feel utterly alone. It is as if all hope has left my body. I place my right index finger on the touchpad. Slowly I move the little white arrow until it lands on the red icon that looks like an old fashioned telephone receiver. I lift my finger and pause for a brief moment before I let it fall to hit the touchpad. Hard. Disconnect. We are disconnected.

## Uncanny Digital Disturbances

Everything in the narrative about the video from Kampala seems to be somehow connected to the use of digital technologies. Thus, digital technologies not only made the video from Kampala available, it was also digital technologies that allowed it to be shared. Similarly, it was also digital technologies that made the connection between the two friends possible in the first place, just as it was digital technologies that finally allowed the connection between them to be brutally severed in the end. Furthermore, the narrator’s emotional reaction might be considered a particularly unfortunate side effect of the narrator’s ignorance about the mechanisms that control what we see on social media. Unaware of what the self-learning algorithms behind the user interfaces do when they select “what is most relevant from a corpus of data composed of traces of our activities, preferences, and expressions” (Gillespie, 2014, p. 168), the narrator fails to comprehend where the real problem lies: not with the 479 friends who failed to acknowledge the post, but rather with the specifications of the algorithm that grants and restricts access to what is posted.

In addition to the point just mentioned, based on this narrative account, there is also another rather more curious point that remains to be made, which illustrates how digital technologies can affect our way of making sense of the Self, the Other, and the World. Indeed, when the narrator is confronted with a kind of digital doppelgänger (Cleland, 2008), the narrator is overwhelmed by a feeling of uncanniness in the Freudian sense of the word in which the uncanny is used to refer to “that class of the terrifying which leads back to something long known to us, once very familiar” (Freud, 1925/2003, p. 124). Surely, the idea of having a doppelgänger is uncanny in itself, but when another further presence is also introduced, we might imagine the alienating feeling of the uncanny to be exacerbated, doubled, multiplied. From the narrator’s point of view, it might feel as if her doppelgänger has invaded the privacy of the other person’s home, after which the strange specter has decided to join the conversation. In one sense, Foucault’s (1986) description of the experience of seeing one’s own image reflected in a mirror resembles the experience of seeing one’s own image on a computer screen. Hence, according to Foucault (1986),

In the mirror, I see myself there where I am not, in an unreal, virtual space that opens up behind the surface; I am over there, there where I am not, a sort of shadow that gives my own visibility to myself, that enables me to see myself there where I am absent. (p. 24)

In both cases, when one’s image is reflected by the mirror or broadcast by the computer screen, the image is suspended in a paradoxical liminal space between the real and the virtual where the mediated mirror image “sets up an ambivalent and oscillating set of responses, it is both ‘me’ and ‘not-me,’ ‘real’ and ‘not-real,’ ‘self’ and ‘other’” (Cleland, 2008, p. 47).

## Making the Family Strange

There are five of us: my mom, my dad, my younger sister, my older brother, and me. When I think of us and think about how others would think of us, I think of us as a traditional nuclear family. Over the last year, however, things have somehow begun to change between us. Not drastically, but enough to be felt. I suspect that my ethnographic fieldwork in Uganda and my reading about postcolonialism and posthumanism might have had something to do with it. I have been deeply affected by my readings and experiences to the extent that you might say that I have begun to live (with) theory.

On day number 57 of the nationwide lockdown, we are casually chatting about whatever each of us has been doing for the last couple of days. And so as we sit there, the five of us, peacefully around the solid surface of the wooden table in my parents’ dining room, we seem to form a perfect tableau. None of us has been doing anything out of the ordinary nor even anything that might be characterized as interesting. And so my sister finally resorts to start reporting on what she and my brother have been watching on Netflix lately. My mom and dad giggle in sync and smile:

That’s funny, we also just finished watching that show.

Even though I feel sick at heart, I try to keep up a cheerful facade while pretending not to feel the slightest bit provoked or alienated by the banality of the conversation. But my mom immediately picks up on my phony act and does what moms do when they sense that something is rotten in the state of Denmark: She bluntly asks without any beating about the bush whatsoever:

“What’s on your mind, my friend?” I try to control my emotions by taking a deep breath before I proceed to declare my answer as if I were an American patriot reciting the pledge of allegiance:

I am planning on going to the Black Lives Matter demonstration on Tuesday!

The exclamation lingers briefly in the air above the wooden table, below the Danish designer lamps from the 1970s that are common in the homes of the educated middle-class families. In my head, I finish my potent proclamation eloquently by silently adding,

With liberty and justice for all!

My answer sends shockwaves through the room and suddenly causes a rapid process of metamorphosis to ensue. My dad tries to keep his voice calm, but he is clearly outraged:

Why do you want to participate in a demonstration for a man who lost his life in America? Do you really think that it is a good idea during this pandemic?

My brother seizes the chance to vent his indignation while also capitalizing on the opportunity to come across as knowledgeable, rational, and enlightened:

“I *really* think it’s a horrible idea! Imprudent even!” He says in a supercilious tone of voice that makes me feel like a child being scolded unreasonably hard for a misdemeanor violation of some kind. I feel the color of my face turn from its usual pink to crimson.

“At 30 years old, I really thought you were smarter than that,” he says and makes a haughty snort before he continues in a particularly condescending manner:

But Listen! You need to listen to me! You need to put things in perspective!

I stay silent, but on the inside, I am boiling with anger and resentment. My brother, who seems to be encouraged by the sound of his own voice, continues, now practically spitting the words in my direction:

I’m really offended that some people can act so selfishly!

I remove a drop of something from my left temple and wonder if it might be my own sweat or if it is perhaps my brother’s saliva. Even though I have stopped listening, I fail to dodge his final comment:

Have you even thought about grandma?! You might just end up killing her if she gets infected from you!

I try to keep my cool, but my voice breaks as I make a feeble attempt to pronounce the very first syllable of the sentence:

Fi-irst of all, the man did not just lose his life—he was brutally murdered! And second, I really think it is important to support people who dare to stand up against an oppressive system.

I am literally on the brink of crying. I feel the pressure of tears, making their way up and into the balls of my eyes, but at least I manage to stutter my way through the next sentence and succeed in delivering my main point:

To-to lis-listen to them and realize that we are also living in an oppressive system with lots of structural racism here in Denmark.

My dad, always the diplomat, tries to meet me halfway:

I agree with you, I do, and I really think it’s an important issue, really! And I would have participated myself had it not been for corona . . . and for the fact that mom and I have to go and get a new greenhouse for the garden on Tuesday.

## Digital Differences

Before the corona pandemic, one might have entertained the idea that a virus such as corona would affect everybody to the same extent and in the same way. Be that as it may, just as other threats of environmental collapse impact different people differently, so the pandemic has made it clear that the various classes of humanity are affected differently by the coronavirus depending on such things as country of origin, color of complexion, and sexual characteristics.

Corona is yet another telling sign that tells us how inequity pervades the way in which most societies are structured. In support of this point, Bignall and Braidotti (2019) state that while the consequences of the Anthropocene are planetary, the Anthropocene results in “a culturally and biologically differential experience, inflected by diverse materialities and vitalities” (p. 14).

Not only does the virus not affect different people in the same way, because there is a close connection between corona and the use of digital technologies and because different people have different possibilities for accessing various types of digital technology, our experiences may vary a great deal depending on the kinds of digital technologies we prefer and use. This, in turn, in addition to the fact that digital technologies also compete among each other for our attention (Kalpokas, 2019), results in different people having different experiences of what is happening in the world around them. Thus, the misunderstandings described in the narrative about the family conflict may be understood, we suggest, not necessarily as a result of fundamentally different worldviews, but rather as a result of the different ways in which the narrator and the narrator’s parents consume information via digital technologies and social media in different ways.^1^

## Troubling Technologies

There might have been a time not too long ago when many of us imagined that technologies such as virtual reality, for example, would ultimately liberate humankind from the restricting constraints of culture, history, tradition, and, yes, perhaps even from our own bodies thereby freeing us from our human nature so that we might finally be allowed to impact the future and transcend the present (Pillow, 2003). That vision has not been realized, however. As it turns out, our new reality with corona, characterized most prominently by the ubiquitous presence of and dependence on digital technologies and social media, has not had the liberating effect many had hoped for. Nor has the possibility of creating alternative identities offered by social media. And so, digital technologies, such as virtual reality, have not set us free. We cannot be whatever we want to be. We can only be whatever the clever algorithm behind the user interface allows us to be! In fact, with the onset of corona and the increased use of digital technologies, we might, in fact, have come to feel *more* restricted due to the way our daily lives are structured and sometimes hampered by the build-in characteristics of particular digital technologies and what they allow us to do. In keeping with this idea, even if we wanted to, we cannot very well escape the influence of digital technologies. First, they keep us from logging out metaphorically speaking because of the addictive inevitability (Markham, 2020) with which they have invaded all aspects of our lives (cf., Berry, 2020; Gilroy-Ware, 2017), and second, if we were to somehow succeed in logging out, for example, by forsaking social media, we run the high risk of excluding ourselves from a number of communities (Pangrazio, 2019), not only in the virtuality of social media but also in the “real” world where it would be hard to interact with others who have not chosen to limit their use of social media and proceed to talk about what goes on there in the real world. *Logging* out would then, in effect, amount to *locking* oneself out (Gangneux, 2019).

According to Barad (2007), “we are *of* the world” (p. 185), and it follows from that that we have always been cyborgs (Clark, 2003; Haraway, 1991). There might nevertheless be good reasons for reemphasizing and reconsidering our cyborg nature in light of our dependence on digital technologies, especially because of the agential nature of these digital technologies and their ability to affect us and each other in profound ways (Warfield, 2016).

## Blue Skies

The cursor flickers mercilessly. Even the slightest pause and there it is with its incessant flickering. As if it is waiting impatiently for me to resume writing. Tap. Tap. Tap. Come on! Get on with it! But I don’t know what to write. Is this really supposed to be so damned hard? I think to myself while staring at the blank screen of my laptop. To be honest, it feels absurd to sit comfortably at home during a global pandemic, reading, and writing and responding to the second of 21 prompts meant to inspire reflection and spark creativity as part of a call for participation in a project about life in the time of corona (Markham et al., 2020). But I *am* responding. And I *do* sit comfortably at home. Others have responded by posting pictures of their cats, coffee mugs, and computers to answer today’s prompt. I can do that. I have a cat, a coffee mug, and a computer. But what would that accomplish when lots of other people have already written thoughtful texts with profound insights from the perspective of cats, cups, and computers?

Just as I am about to quit, my computer saves me from the dreary stalemate by informing me that it needs to be rebooted. I don’t know why it suddenly feels like that. However, as I can easily relate to its desire for a chance to begin again and anew—I sure wish I could reboot myself as well—I decide to fulfill its wish and follow the instructions for how to reboot while keeping my gaze firmly fixed on the screen as if hoping that because of the close connection between me and this device, rebooting the computer will have similar effects on both our systems so that we will both be able to start over from a point of departure less cluttered by all kinds of irrelevant rubbish.

When I hit the tab that makes the computer reboot, the screen turns completely black and then for a brief moment, and for reasons I cannot explain, it suddenly turns bright blue before returning to black once again. The blue color sticks to my retina, and I keep seeing it in my mind’s eye long after it has disappeared from the screen. And suddenly, the memory returns.

The blue color was that of the clear skies above us when we stood in silence outside the whitewashed church of a nearby parish on a Wednesday morning in May. Because of corona, we were forbidden from entering the church, but two enormous speakers had been installed next to the doorway of the church porch. At 11 o’clock, the church bells started ringing. The sound was ear-splitting. When the first part of the ringing was over, I counted the nine angelus strokes in my head in fearful anticipation of what I knew was next on the agenda: Singing! As if on cue, the tears started running the moment when the first note of the organ poured out of the enormous speakers. The cantor had a deep yet piercing voice that made my body shake and sob uncontrollably.

Nearer, my God, to thee,

Nearer to thee!

I tried to sing along, but no words came from my mouth. They got stuck in my throat and would not come out no matter how hard I tried. The only sound I made was a strange stuttering sound of staccato breathing that I could not stop myself from letting out.

E’en though it be a cross

That raiseth me.

I grabbed my husband’s hand and squeezed it as hard as I could. He squeezed mine back as if the pain of squeezing and being squeezed would somehow affirm the fact that unlike the body inside the white casket, we were still alive, the two of us, together, not forever, but for now. I knew that he was crying too. And still, the sun kept shining from those clear blue skies. It felt wrong! Why did it not rain? There was no reason for the world to be smiling. No reason at all. And I certainly did not want to be nearer to God. I was angry with God. To hell with God!

When the service was finally over, and the bells started ringing once again, the verger opened the heavy double doors. Again, we waited in the deafening silence that followed in the wake of the ringing bells, dreading once again what we knew would happen next. Even if no more than a minute or two passed before the shadow of the pastor in her black robe appeared in the doorway, the pent-up tension made it feel as if time had stopped its relentless forward movement toward the unknown of the future. The pastor was followed by six pallbearers in two lines of three, carrying the white casket containing the body of the deceased. At the sight of familiar faces, some disfigured from crying, others from the unbearable exertion of trying to refrain from crying, my own violent crying resumed until finally, I could take it no more when confronted with the sight of the two little figures of sobbing grandchildren trailing behind the casket with signs of confusion and despair painted all over their innocent little faces. Their lavender dresses seemed to sparkle in the bright sunlight in stark contrast to the slow-moving sea of darkness formed by the uniform black attire of the adults around them. They clasped each others’ hands. Tears ran down their faces. In the opposite hand, each of them held a single red rose that somebody had given them and which was to be placed on the casket as a final farewell. I did not see the children perform this last symbolic gesture. I couldn’t bear to look, and so I turned my head and looked away.

I move my head as if remembering this situation forces me once again to look away. The physical movement awakens me, and as I turn my head back, I am transported back to my study, and I once again find myself seated behind the desk, in front of the laptop, once again confronted with the insistent flickering of the impatiently waiting cursor. But my time travels have inspired me. I know what to write now. My fingers dance across the keyboard as the words begin to flow: “The date is March 17, 2020. It’s a Tuesday. The corona pandemic is wreaking havoc across all continents except in Antarctica, as far as I know. Penguins apparently are immune to its deadly effects.”

## Notes

1. We are well aware that some readers might feel frustrated by the fact that we apparently seem to ignore the central theme of this narrative account by neglecting to discuss the systemic racism expressed in and through the dialogue. Nevertheless, we deliberately leave it as is, first, because this paper is not about racism rather its aim is to cast light on how digital technologies influence our lives in the time of corona, and, second, because we believe that even without an explicit discussion, this narrative account fulfills its performative potential if it succeeds in affecting the reader to critically examine their thinking about racism and related issues.
